# Supplementary material for: Gender roles in ruminant disease management in Uganda: Implications for the control of peste des petits ruminants and Rift Valley fever
Source: PLoS One. 2025 Apr 25;20(4):e0320991. doi: 10.1371/journal.pone.0320991 (PMC12027259; doi:10.1371/journal.pone.0320991)
Supplement: S1 File — (DOCX) [file pone.0320991.s001.docx]

**Focus Group Discussions with adult men and women Livestock keepers (small and large ruminants)**

**Objectives**

The objective of the study is to understand more about the resource base for livestock rearing in the communities, their gender roles in ruminant production in different systems and benefits of Small/Large ruminants (SR/LR). The knowledge of men and women about SR/LR diseases and disease coping strategies will be determined as well as knowledge of risky practices/behaviors leading to exposure to zoonoses (diseases transmitted from human to animals and vice versa) such as RVF. Participants’ views on animal health services within their community and systemic gender constraints faced will be explored. The information from this study will be used to inform strategies and options for controlling PPR and RVF.

**Methodology**

Various participants for the focus group discussions (FGDs) will be identified and selected purposively in consultation with local leaders and Community Animal Health workers in the Project sites where intensive studies will be conducted for PPR (Nakapiripit, Serere, Isingiro) and RVF (Sembabule, Isingiro, Butebo, and Napak). Participants will be drawn from male household heads (MHH), women in male headed households (WMHH) and women household heads (WHH). The selection of FGDs will be based on those who are actively involved in ruminant production in their communities. A courtesy visit to the local leaders will be made to inform them about the study objectives and the way the study will be conducted. Different participatory rural appraisal (PRA) methods will be used including simple ranking, seasonal calendar, resource mapping and proportional piling. The discussion will explore perceptions of men and women on the village resources for ruminant rearing, disease impact, knowledge on ruminant diseases, role of men and women in disease management, the risk factors contributing to occurrence of the diseases, risks of exposure and the extent to which they access the animal health services available in their communities. Separate FGDs will be held with adult men, adult women between the ages (18-60years). Each FGD will have between 6-8 participants. Two sub counties per district and two parishes per subcounty will be selected. The group sessions will last for 90-120 minutes. The objectives of the study and the benefits will be explained to the participants prior to the discussions so as they make an informed decision to participate (or not) and permission to record the discussions will be sought from the participants. A suitable location will be identified for the discussion and two researchers from ILRI will facilitate the discussions: a facilitator / note taker and a translator who understands the local language in the study site. The facilitators will seek permission from the participants to capture the discussions both in writing and audio recording beforehand as well as permission to take photographs of the focus group. For all FGDs, informed consent will be obtained from all the participants through signage or thumbprinting.

**Focus Group Discussion Guide:**

**Introductions**

-Introduction of participants and facilitators

-Setting ground rules

**Icebreaker**

-Please tell us about this village/community and the major types of livestock reared? Which livestock are majorly owned by women/men in this community? Why?

**SECTION A:** **resources for livestock rearing, priority diseases and Animal health services**

| **SN** | **Guiding Question (Facilitator)** | **Process (Facilitator)** | | **Output** | |
| --- | --- | --- | --- | --- | --- |
| **A** | **key resources for sheep and goat rearing, access by gender** | | | | |
| **1a** | *What important natural resources do you have for rearing SR/ large in your community? Who uses them and how? Where are these resources located?* | Men and women in separate groups map out the key livestock resources on a large sheet of paper that are important to them for livestock rearing (grazing areas, watering places,  animal health services, livestock markets, trade routes, slaughtering sites  seasonal movements of flocks and wildlife areas)-probe for rights of access to men and women, then later the two groups are brought together in a plenary to discuss and compare their results. | | Village resources map for each group drawn and all discussions describing the key features recorded as well as differences/similarities in the maps | |
| **B** | **Knowledge of SR diseases and risks factors** | | | | |
| 2 | What roles/activities do you perform in ruminant production | In separate groups, women brainstorm and make a list of all the roles women and men. Men do the same in the men’s group | | Recorder in each group draws a chart with columns for F/M/Both. | |
| 3a. | *What are the common disease problems that affect your SR/LR?*  *Probes: Which age groups are most affected? How would you recognize these diseases (signs of each disease)? How does the disease progress? For how long does it last?*  *What do you think causes these diseases? (probe for causes and understanding of transmission)*  *Which disease problems are transmitted from animals to humans and vice versa? How are these diseases passed from animals to humans and from humans to animals? Who in the household is mostly at risk?* | In separate groups both men and women together list all the SR diseases and the clinical signs for the mentioned disease. Each disease is written on a card in local names and group ranks diseases. (disease with highest mortality first). Let the farmers indicate the disease in the local language, what clinical signs and actions taken in case of disease occurrence | | Results are recorded and all discussions during the ranking | |
| b | How do you manage these diseases?  What treatment do you use? (modern medicine, tradition) how effective are these? Where do you get advice from?  What do you do to avoid your animals getting sick? | In separate groups, men and women list actions taken by each gender in response to diseases (probe for reasons in the differences in disease management [if any] | | Responses recorded for f/m/both | |
| 5 | If you found one of SR/LR dead what would you do? (handling practices, hygiene) |  |  |  |  |
| 6 | *Which months of the year do you mostly have these diseases and high mortality? What are the major factors that give rise to these diseases?*  *What activities do you think can expose you to livestock-human disease infections?* | In separate groups, men and women mark the seasonal divisions of the year along the top of the line drawn on the ground, starting with the rainfall calendar and process repeated for other issues (management activity, event, disease) one calendar under the other probing to understand when the diseases seem to occur i.e. during which season and which species is affected most | | Separate seasonal calendars drawn to visualize the perceptions of seasonal variations in livestock practices, diseases, risk factors and mitigation measures  Discussions on the differences are recorded | |
| 7 | What do you do to minimize the risk of livestock disease infection to humans in your households? (Production strategies like changing herd composition, practices including sanitation or quarantine, veterinary interventions including drugs, traditional medicine, vaccines) |  |  |  |  |
| 8 | In your opinion, what could be done to increase the awareness of RVF and associated risks of human infection in your community among men and women livestock keepers | Facilitated discussion | | All discussions recorded | |
| **C** | ***Women’s and men’s perceptions of animal health services*** | | | | |
| 9a. | Which organizations or individuals in your community provide vet/animals services?  If someone wanted vet drugs/vaccines where would they go? Why? | | In separate groups of men and women, participants map out existing animal health services/agrovet shops in their community. Starting from identifiable reference points participants mark out the locations of these services on a large sheet of paper, with different colored cards of different sizes prepared in advance to indicate the most important and accessible services to them and the ones where they are excluded and the reasons for that.  Participants indicate what they would like to see in their community that is currently not on the map. | | Record results |
| b. | How many vets/Animal Health workers are men? How many are women? How do they relate with women? | |  |  |  |
| c | How do you get to know about which vaccines are for the major disease? (e.g: trainings, extension, information campaigns, advertising of mass vaccination days) Who is most likely to receive this information (men, women, youth, and location)? Why?? If you are to share this type of information which people would give it to? Why? | |  |  |  |
| d | What factors would hinder a female/male livestock keeper from accessing veterinary services in your community?  e.g. financial resource, constraints, mobility, etc. probe for those that seem more socio-cultural | | In separate groups of men and women, participants list all the constraints that prevent them from accessing veterinary services (veterinary drugs, vaccines, trainings etc. The constraints listed are grouped and clarified. Is the problem social cultural, financial (availability Vs affordability) or institutional? Probe for the causes of each constraint and how they cope?  How can each constraint be solved? How do the constraints compare for men and for women? | | Recorder draws a template with columns for constraint, causes, coping strategies and solutions. All constraints listed are written on a flipchart as they are mentioned and all discussions around each constraint recorded |
| e | Given the constraints, how could the animal health services be made more accessible to women? | | Facilitated discussion | |  |
| 10 | *How are vaccinations for SR/LR organized in your community? -*What perceptions and beliefs do you have about the vaccination of small ruminants? | | Facilitated discussion-probe for SR/large ruminants diseases most vaccinated, how often it done, who mostly initiates it and how is it done? Is it free? If not, what are the costs? Probe whether there has ever been any vaccination done for PPR/RVF in the past year in their community, how was it done, who provided it? Who accessed it? Why? | | Responses recorded |
| 11 | *How could vaccinations for SR/LR be improved in your community?* | |  |  |  |
|  | ***Closure***  -If you could change anything about the current animal health delivery system what would you change? Why?  -Is there anything else you would like to discuss that we did not ask you? | |  | |  |

**SECTION B. LIVELIHOODS AND MARKETS**

The activities under section will be done only in the PPR districts with different FDGs from the ones above but held in the same sub counties.

**Objectives**

The objective of this section is to understand the sources of livelihoods for men and women in the targeted communities and how the small ruminants fit in. Also, to try and understand the dynamics around the marketing of small ruminants in this community and the different sources of vaccines/vet drugs for each gender and their delivery modes.

**Methodology**

Consent will be sought before the farmers are interviewed. The farmers will be separated in women and men’s groups, each group between 6-8 members. Each group will be asked the same questions and then they will be brought back to plenary to discuss and agree on some of the key issues.

| **SN** | **Guiding Question (Facilitator)** | **Process (Facilitator)** | **Output** |
| --- | --- | --- | --- |
| **A** | **Livelihoods sources** | | |
| **1a** | What are the major types of livestock reared? Which livestock are most important women/men in this community? Why?  Among the livestock keepers, who are the small holders? Who are the medium holders? Who are the large-scale holders? | In separate groups, men, and women, list the major livestock species that are reared in the community and then rank them in order of importance for each gender. Let them give reasons for each choice. probe why small ruminants are important and inquire how they would categorize smallholder, medium holder, and a large-scale holder in this use the number of does and ewes that are owned. |  |
| **b** | What benefits have you gained from rearing SR? | Get a list of benefits in separate groups of men and women. Using a flip chart, let the group give a list of the main reasons why they keep ruminants in their community. Let them rank the most important in order of priority with reasons. | Template drawn with benefits and rank for f/m/both |
| **c** | What different activities do men and women do to support their livelihoods? (differentiated by social and economic group)  For each socio-economic group, what proportion of income comes from the different livestock species (SR/LR) for meeting the household needs, education, health care? | In separate groups, Women and men brainstorm and make a list of all their livelihood activities. using 100 counters they indicate proportions of the number of different gender groups involved in that activity. The group ranks the livelihoods activities in order of priority in terms contribution to the household’s income, food security and health. (*Note if small ruminants are not part of the list probe to know how they would rank it if it were included in the list, if livestock production is mentioned please continue and break it down by livestock species and also rank in terms of contribution to income health and food security).for those that are indicated for income please probe for what the income is used for* | recorder draws a template with activities for men and for women or both, with rank for each activity and reasons for the rank. |
| **B** | **Sales and flock dynamics** | | |
| **2** | What are the main livestock and livestock products sold in the community? Which type of livestock bring the most money to men/women? | Together men and women draw a seasonal calendar with all the months filled in at the top of the calendar and indicate the periods when sales happen for each type of livestock. probe for reasons why this happens in that period of the year. Probe for who makes the decision to sale man/woman/both— | A completed template showing  important livestock or livestock products sold in order of priority (space for five items) and the time of the year when this happens, who sells them  . |
|  |  | Then display a map of the area (district highlighting their village) to the group. let the group Map out the geographical flow (physical) of small ruminants separately, Indicate the small ruminant production points in the map (district). Indicate on the map where the marketing points are. Facilitate a discussion among participants about the most important animal products that are taken to the markets, whom they sell to in those markets and the prices. Other probes will include any changes in the flow pattern during other parts of the year? | All discussions on the displayed map are recorded |
| **C** | **SR inputs and Vaccine traits** | | |
| **3** | Where do the SR farmers obtain their inputs/vet drugs for goat/sheep rearing? Why would men/women choose to buy/use certain vet drugs/vaccine from over another? Where do you mostly buy these drugs? Why? | Together men and women list the different inputs that are used in SR production.  On the same map used above, the group indicates where the different service providers are located (for the sources of drugs/inputs listed above. The group to list the different animal vaccines/drugs that are stocked in these shops and for which animals. If the district or subcounty offices also stock some vaccines have them mapped. For each of the channels that are indicated list them in the table. Try and work out the cost of delivering each of the vaccines that are indicated to their area from each source. Also indicate the cost of buying the vaccine. Ask the farmers what the cost would be if the vet came to vaccinate versus them buying and administering it themselves. Ask the group if they have ever used any of the vaccines mentioned above and for which livestock, and the prices and challenges faced in accessing the different inputs.  Again, facilitate a discussion with participants about the characteristics they would consider important before choosing a vaccine to apply to their animals. (characteristics of the vaccine characteristics of the delivery system). |  |
|  | Closure | Is there anything else you would like to discuss that we did not ask you? |  |
