# Supplementary material for: Gender roles in ruminant disease management in Uganda: Implications for the control of peste des petits ruminants and Rift Valley fever
Source: PLoS One. 2025 Apr 25;20(4):e0320991. doi: 10.1371/journal.pone.0320991 (PMC12027259; doi:10.1371/journal.pone.0320991)
Supplement: S2 File — (DOCX) [file pone.0320991.s002.docx]

**Key Informant Interview Guide**

**General information**

The purpose of the study is to understand the role women and men in small and large ruminant (SR/LR) production in terms of disease management and control in targeted project sites. The interviews will be conducted with; **-Animal health professionals** (Veterinarian, Animal husbandry officer or Livestock officer, community animal health worker (CAHW)/Vet Scouts, surveillance focal person/a Member of the Disease surveillance committee),

**-Policy makers/Researchers** (national staffs from the Ministry of Agriculture, Animal Industry and Fisheries (MAAIF) /researchers and other NGOs) with knowledge about the implementation of animal health services and vaccination programs across the project Districts, District Veterinary Officers and District Community Development Officers.

**-Vaccine distributor** (employee of an agro-veterinary shop (agrovet) selling vaccines or someone working in the procurement of vaccines in a national or private laboratory, or someone involved in any way in the distribution of livestock vaccines such as the DVOs, CAHWs

**-Local leader** – can be a Local Community leader (Parish chief, Sub county Chief, Local council 1 chairman or community development officer, opinion leader, or a farmer group leader/ a herd leader in the case of pastoralists who is active in the community and knowledgeable about the opinions and practices of fellow SR/LR farmers

| Name of Interviewer |  |
| --- | --- |
| Location/District |  |
| Interviewee title |  |
| Gender |  |
| Time interview started |  |
| Time interview ended |  |

**Duration:** 25 minutes

**Introduction** (2 minutes)

- Welcome—Explain purpose of the interview and lay out potential risks and sensitive information and how they will be handled. Explain the benefits of participating in the research study and give contacts of the research team for further follow up.

Thank you for agreeing to do this interview. My name is [NAME OF RESEACHER] and I am working for ILRI an international research organization that focuses on livestock research and development. I will be talking with you today and the interview will focus on four key areas: knowledge of SR diseases, access to services, systemic gender constraints, and impact of disease. This study is part of the BUILD Uganda project funded by BMZ implemented by ILRI in partnership with MAAIF. I would like to hear your views and experiences as regards the roles of women and men in Small Ruminant (SR) production, exposure to risks through contact with sick animals and mitigation measures used. The information collected from this study is for research purposes only and will inform our project’s interventions/ strategies in controlling PPR and RVF. The interview will last about 25 minutes.

- Everything you say will be confidential. To protect your privacy, your name will not be associated with anything that you say or to any reports of this study. Besides, the telephone number and your name I have from the list of key informants will be kept confidential and rather an identification code will be assigned to the notes.
- At any time during our conversation, please feel free to let me know if you have any questions or if you would rather not answer any specific question You can also stop the interview at any time for any reason.
- Please remember that we want to know what you think and that there is no right or wrong answers.
- Ask for verbal consent*.*

**Role and experience** (3 minutes)

Drawing on your personal experience as a [TITLE] in [LOCATION], I would like to begin by asking you some questions about your current role as it relates to working with livestock keepers.

- What are your major responsibilities in your current position?
- Please tell me a bit about your experience working with livestock keepers more especially SR/LR and animal health care services?

1. (**For women**) What is it like to be a woman working as an animal health professional/vaccine distribution? Challenges? Advantages? Are there any different expectations that the community/has of you because you are a woman? What portion of your colleagues in this position are women?
2. **For men**) what portion of your colleagues in this position are women? Why do you think this is? Barriers or advantages to being a woman in this profession? Are there any different expectations the community has for women in this role?
3. **(For local leaders)** For how long have you been living and working in this community? Do you keep livestock yourself? What type of livestock do you keep?

**Small/Large ruminant production, challenges faced and exposure to zoonoses** (10 minutes)

A key goal of the BUILD project is to support ongoing campaigns to eradicate PPR and control zoonotic diseases. I would like to get your opinions about the roles played by women and men in SR/LR production and in the control of livestock diseases.

1. What type of livestock are majorly owned by women in the in the community you serve?
2. What role do women play in small/large ruminant production? For example, what types of work do they do? How do they benefit? How do women’s goals differ from those of men with regards to keeping small/large ruminants?
3. What are the major challenges for small/large ruminant keepers in the communities where you work?
4. How do men/women manage ruminant diseases? Are there any differences in the way men and women manage ruminant diseases? Under what circumstances do women make decisions about veterinary care?
5. What major livestock diseases have caused infections to human in your community? Who has been mostly affected? Why?
6. What do men/women do in case of sudden death of an animal? (handling practices, hygiene)
7. What behaviors/activities if at all, have you observed among men/women livestock keepers that could expose them to livestock-human disease infections? Is it the same for men and women? Different?
8. How do men perceive the risk associated with these behaviors/activities? How do women perceive the risks?
9. What measures do men/women take to minimize the risk of livestock disease infection (livestock to human infection? (Production strategies like changing herd composition, practices including sanitation or quarantine, veterinary interventions including drugs, traditional medicine, vaccines)
10. In your opinion, what possible strategies could help improve the awareness of RVF and minimize on the risks associated with human infection in your community among livestock keepers?

**Animal health services (5 mins):**

One of the outputs of the BUILD project is that livestock keepers understand the benefits of vaccination and use it for selected diseases (PPR and RVF). I would like to understand more about how vaccinations are organized in the communities

1. How is vaccination for PPR/RVF organized in your community/where you work? Specifically, how is information on vaccination disseminated? (types of messages/medium are used, who is involved, who participates)? How effective is it? What perceptions and beliefs exist among men and women about the vaccination of small ruminants?
2. What challenges do women face in accessing vaccination services (socio economic, cultural)? How are women who access them perceived? Which individuals in your community mostly work with women? Why?
3. What groups or individuals in your community help livestock keepers when their livestock are sick?
4. How is information about vaccines shared with livestock keepers? (Eg: trainings, extension, information campaigns, advertising of mass vaccination days) Who is most likely to receive this information (men, women, youth, and location)? Why?
5. What differences do you see in the way men and women engage with PPR/RVF vaccination?
6. **(For Policy makers/Researchers)** How is PPR/RVF vaccination structured in Uganda? (Is PPR notifiable disease? Public? Private? Mass vaccination?)

Is there a PPR strategy in place? If not, and still under development, how far has it reached? What is contained within? What challenges have you faced while developing it? What improvements would you want to see given the challenges you have faced?

How have previous outbreaks in your area been controlled? What strategies do you have in place to control outbreaks from recurring (community, national, district)?

1. What barriers exist that influence access of vaccination services by women? (Social, cultural, infrastructural barriers?) How are women who use vaccines perceived? Which individuals and institutions work most closely with women in addressing animal health issues?
2. **(For the agrovets/Drug Stockists)** Among the vet drugs/vaccines you stock, do you sell for both small and large ruminants? For which diseases? where do you source these vaccines from? what are the prices? How do you determine the prices for each vaccine? How do you deliver the vaccines to the farmers? Among men and women, who are your main customers? Why? What challenges do you have in selling these vaccines and how do you overcome them?
3. . Of these conditions what would inform your pricing of these vaccines. Could you give us some indicative prices that you think will be favorable for such a vaccine for your clients and you.
4. In your view what characteristics of vaccine would be desirable and acceptable by men/women?

**Recommendations and strategies for engaging SR/LR in control of PPR/RVF (3 mins)**

1. Given the existing barriers influencing access to vaccination services, what recommendations would you suggest to increase women’s access to PPR/RVF vaccines and other animal health services?
2. (**For vaccine distributors/agrovets/local leader)** What strategies would help you to increase access to PPR/RVF vaccination for men and women? What additional resources would you need to support these activities within your current role?
3. **(For policy makers)** What other possible research areas do you think would contribute to realistic and actionable policy recommendations in the communities?

**Closing (2mins)**

-Is there anything else you want to share or mention about any of the topics that we have discussed or other areas that we did not discuss but you think are important?

-Do you have any contacts who you think might be useful and willing to tell us more about this topic?

-Thank you for your time and participation in this interview. The information that you provided to us will be very helpful in this project.
