# Supplementary material for: Gender roles in ruminant disease management in Uganda: Implications for the control of peste des petits ruminants and Rift Valley fever
Source: PLoS One. 2025 Apr 25;20(4):e0320991. doi: 10.1371/journal.pone.0320991 (PMC12027259; doi:10.1371/journal.pone.0320991)
Supplement: S3 File — (DOCX) [file pone.0320991.s003.docx]

Coding framework used in NVivo

Themes

| Name | Description |
| --- | --- |
| diseases | diseases that commonly attack cattle |
| awareness of RVF | awareness and knowledge of zoonoses |
| causes of spread | how the diseases are spread with farms and between farms |
| mortality | age groups most affected |
| occurrence | months when these diseases seem to occur |
| preventive and control measures | how the diseases are prevented or controlled from spreading |
| men | what men do to prevent diseases |
| women | what women do to prevent diseases |
| signs | how the cattle diseases are recognised |
| feeding practises | this includes watering and grazing |
| grazing | where cattle are normally taken for grazing |
| watering | places where farmers get drinking water for their animals or take animals for watering |
| Bore holes | sources of water for the animals |
| Dams | These are water points created for watering animals |
| swamps | for watering animals |
| gender roles | roles/activities performed by men and women in cattle production |
| children | roles performed by boys/girls |
| men | roles performed by men in animal rearing |
| women | roles performed by women in animal rearing |
| influencing factors | sources of animal health care services, accessibility, |
| hindrances | factors that hinder men/women from accessing vet services |
| information | sources of information on veterinary/vaccination services |
| livestock types | common livestock kept by men and women |
| men |  |
| women |  |
| Markets | Marketing outlets for cattle and cattle products |
| Recommendations | what can be done to increase awareness of diseases/zoonoses/improve access to animal health services |
| Risky practices | practises, behaviours, activities that expose them to zoonoses |
| prevention | how they minimise the risk of infection |
| vulnerability | who is most at risk in the household |
| Prevention and control options | vaccination/spraying areas |
| accessibility | most accessible and inaccessible services |
| vaccination | commonly vaccinated diseases, frequency of vaccination, perceptions on vaccination |
| barriers and perceptions | Views on vaccination of cattle |
| recommendations |  |
| vector control practises | common vectors, challenges faced |
| challenges |  |
| recommendations |  |
| vectors |  |
