# Supplementary material for: Gender roles in ruminant disease management in Uganda: Implications for the control of peste des petits ruminants and Rift Valley fever
Source: PLoS One. 2025 Apr 25;20(4):e0320991. doi: 10.1371/journal.pone.0320991 (PMC12027259; doi:10.1371/journal.pone.0320991)
Supplement: S1 Table — (DOCX) [file pone.0320991.s004.docx]

**Table 1: Levels of themes from the analysis of factors influencing disease control option uptake by gender and production system**

| **Ecological level theme** | **Subtheme** | **Cattle** | | | | | |  | **Small ruminants** | | | | |
| --- | --- | --- | --- | --- | --- | --- | --- | --- | --- | --- | --- | --- | --- |
|  |  | **pastoral** | | **agropastoral** | | **mixed** | | **pastoral** | | **agropastoral** | | **mixed** | |
|  |  | **M** | **W** | **M** | **W** | **M** | **W** | **M** | **W** | **M** | **W** | **M** | **W** |
| **Intrapersonal level** | Fear of vaccine side effects | 9 | 2 | 5 | 7 | 0 | 3 | 5 | 4 | 0 | 0 | 2 | 0 |
|  | Decline in milk production | 0 | 0 | 1 | 1 | 1 | 1 | 0 | 0 | 0 | 0 | 0 | 0 |
|  | Milk/meat consumption suspended | 1 | 1 | 1 | 0 | 2 | 2 | 0 | 0 | 0 | 0 | 0 | 0 |
|  | Disinterest | 0 | 1 | 0 | 0 | 0 | 0 | 0 | 0 | 0 | 0 | 0 | 0 |
|  | Resistance to diseases | 2 | 0 | 0 | 0 | 2 | 2 | 2 | 0 | 1 | 1 | 0 | 0 |
|  | Fear of income loss | 0 | 0 | 0 | 0 | 0 | 1 | 1 | 1 | 0 | 0 | 1 | 0 |
|  | Limited knowledge on diseases | 0 | 1 | 0 | 2 | 0 | 0 | 0 | 1 | 0 | 0 | 0 | 1 |
|  | Mistrust of   vaccines | 0 | 0 | 2 | 0 | 0 | 1 | 0 | 0 | 0 | 0 | 1 | 0 |
| **Interpersonal level** | Festive seasons | 0 | 0 | 0 | 1 | 0 | 0 | 1 | 0 | 0 | 0 | 0 | 0 |
|  | Mistrust of animal health workers | 0 | 0 | 0 | 2 | 0 | 0 | 0 | 0 | 0 | 0 | 0 | 0 |
|  | Rumors from neighbors | 0 | 0 | 0 | 0 | 0 | 1 | 1 | 0 | 0 | 0 | 0 | 0 |
|  | Domestic chores | 0 | 3 | 0 | 1 | 0 | 2 | 0 | 0 | 0 | 0 | 0 | 0 |
| **Institutional level** | Vet service costs | 1 | 4 | 2 | 2 | 1 | 2 | 2 | 2 | 2 | 2 | 2 | 2 |
|  | Lack information on vaccination | 3 | 4 | 2 | 2 | 1 | 2 | 0 | 0 | 0 | 0 | 0 | 1 |
|  | Distance to vaccination centres | 2 | 2 | 0 | 2 | 0 | 1 | 2 | 2 | 2 | 1 | 1 | 2 |
|  | No cattle crushes (key infrastructure) | 0 | 0 | 0 | 1 | 2 | 0 | 0 | 0 | 0 | 0 | 0 | 0 |
|  | Poor quality vaccine | 0 | 1 | 3 | 2 | 0 | 1 | 0 | 0 | 0 | 0 | 0 | 0 |
|  | women taken as minority by extension workers | 0 | 2 | 0 | 0 | 0 | 0 | 0 | 0 | 0 | 0 | 0 | 0 |
| **Community level** | Information vaccination outreaches | 0 | 0 | 0 | 1 | 0 | 0 | 0 | 0 | 2 | 3 | 1 | 2 |
|  | Fear of disease spread | 0 | 0 | 1 | 0 | 0 | 0 | 0 | 0 | 0 | 0 | 0 | 0 |
| **Policy Level** | Insufficient vaccines | 1 | 1 | 4 | 3 | 2 | 2 | 1 | 0 | 0 | 0 | 0 | 0 |
|  | Few AHW | 0 | 0 | 1 | 1 | 0 | 1 | 2 | 2 | 0 | 1 | 2 | 1 |

Notes:  numerical number = Number of FGDs mentioning a factor, M=men, W=Woman, AHWs=animals health workers

The vaccine side effects mentioned included: fear of animal death, abortions, tail fall off, sickness in animals/overdose, bareness, limping/ lameness, restlessness neck swelling, blindness

Mixed = refers to mixed-crop livestock production system
