# Supplementary material for: Gender roles in ruminant disease management in Uganda: Implications for the control of peste des petits ruminants and Rift Valley fever
Source: PLoS One. 2025 Apr 25;20(4):e0320991. doi: 10.1371/journal.pone.0320991 (PMC12027259; doi:10.1371/journal.pone.0320991)
Supplement: Attachment — Submitted filename: Response to Reviewers.docx [file pone.0320991.s006.docx]

*April 12, 2024*

*Dear* *Dr Nussieba A. Osman,*

*We would like to thank you for giving us the opportunity to submit a revised draft of the manuscript “Gender roles in ruminant disease management in Uganda: Implications for the control of peste des petits ruminants and Rift Valley fever. We appreciate the time and effort that you and the reviewers dedicated to providing feedback on our manuscript. Our responses to the reviewers’ comments are in italics below and the changes are highlighted within the revised manuscript. Where line numbers are indicated, they refer to the revised manuscript (Track changes mode).*

**Comment 1:**

Please ensure that your manuscript meets PLOS ONE's style requirements, including those for file naming. The PLOS ONE style templates can be found at

<https://journals.plos.org/plosone/s/file?id=wjVg/PLOSOne_formatting_sample_main_body.pdf> and <https://journals.plos.org/plosone/s/file?id=ba62/PLOSOne_formatting_sample_title_authors_affiliations.pdf>.

*Response: Thank you for pointing this out. The file naming has been revised as per the PLOS ONE’s style requirements.*

**Comment 2:**

Thank you for stating in your Funding Statement: “This work was supported by the German Federal Ministry of Economic Cooperation and Development (BMZ) through the project Boosting Uganda’s investment in livestock development (BUILD) (Grant number BMZ001). Additional support was received from the CGIAR Research Programs on Livestock and Agriculture for Nutrition and Health. We also acknowledge the CGIAR Fund Donors”. Please provide an amended statement that declares *all* the funding or sources of support (whether external or internal to your organization) received during this study, as detailed online in our guide for authors at <http://journals.plos.org/plosone/s/submit-now>.  Please also include the statement “There was no additional external funding received for this study.” in your updated Funding Statement. Please include your amended Funding Statement within your cover letter. We will change the online submission form on your behalf.

*Response: Thank you for highlighting this. We have now included all sources of support and added a statement that there are no other additional external funding sources:*

*This work was supported by the German Federal Ministry of Economic Cooperation and Development (BMZ) through the project Boosting Uganda’s investment in livestock development (BUILD) (Grant number BMZ001) and* One Health Research, Education and Outreach Centre in Africa *(OHRECA) (Grant number BMZ002). Additional time support was received from the CGIAR Initiative Sustainable Animal Productivity (SAPLING) which is supported by the contributors to the CGIAR Trust Fund. (*[*https://www.cgiar.org/funders*](https://www.cgiar.org/funders)*). There was no other additional external funding received for this study.*

***Comment 3:***

We note that Figure 1 in your submission contain [map/satellite] images which may be copyrighted. All PLOS content is published under the Creative Commons Attribution License (CC BY 4.0), which means that the manuscript, images, and Supporting Information files will be freely available online, and any third party is permitted to access, download, copy, distribute, and use these materials in any way, even commercially, with proper attribution. For these reasons, we cannot publish previously copyrighted maps or satellite images created using proprietary data, such as Google software (Google Maps, Street View, and Earth). For more information, see our copyright guidelines: <http://journals.plos.org/plosone/s/licenses-and-copyright>.

We require you to either (1) present written permission from the copyright holder to publish these figures specifically under the CC BY 4.0 license, or (2) remove the figures from your submission:

a. You may seek permission from the original copyright holder of Figure 1 to publish the content specifically under the CC BY 4.0 license. We recommend that you contact the original copyright holder with the Content Permission Form (http://journals.plos.org/plosone/s/file?id=7c09/content-permission-form.pdf) and the following text: “I request permission for the open-access journal PLOS ONE to publish XXX under the Creative Commons Attribution License (CCAL) CC BY 4.0 (http://creativecommons.org/licenses/by/4.0/). Please be aware that this license allows unrestricted use and distribution, even commercially, by third parties. Please reply and provide explicit written permission to publish XXX under a CC BY license and complete the attached form.” Please upload the completed Content Permission Form or other proof of granted permissions as an "Other" file with your submission.

In the figure caption of the copyrighted figure, please include the following text: “Reprinted from [ref] under a CC BY license, with permission from [name of publisher], original copyright [original copyright year].”

b. If you are unable to obtain permission from the original copyright holder to publish these figures under the CC BY 4.0 license or if the copyright holder’s requirements are incompatible with the CC BY 4.0 license, please either i) remove the figure or ii) supply a replacement figure that complies with the CC BY 4.0 license. Please check copyright information on all replacement figures and update the figure caption with source information. If applicable, please specify in the figure caption text when a figure is similar but not identical to the original image and is therefore for illustrative purposes only. The following resources for replacing copyrighted map figures may be helpful: USGS National Map Viewer (public domain): <http://viewer.nationalmap.gov/viewer/>

The Gateway to Astronaut Photography of Earth (public domain): http://eol.jsc.nasa.gov/sseop/clickmap/

Maps at the CIA (public domain): <https://www.cia.gov/library/publications/the-world-factbook/index.html> and <https://www.cia.gov/library/publications/cia-maps-publications/index.html>

NASA Earth Observatory (public domain): <http://earthobservatory.nasa.gov/>

Landsat: http://landsat.visibleearth.nasa.gov/

USGS EROS (Earth Resources Observatory and Science (EROS) Center) (public domain): http://eros.usgs.gov/#

Natural Earth (public domain): <http://www.naturalearthdata.com/>

*Response: Thank you again for highlighting the improvements we need to make to our manuscript. The map was generated by one of the co-authors, Marsy Asindu. Shapefiles for this map were adopted by the authors from the Ugandan Energy Sector GIS Working Group Open Data Site using ArcGIS:* [*http://data-energy-gis.opendata.arcgis.com/*](http://data-energy-gis.opendata.arcgis.com/)

*The caption for fig 1 has been added as follows: Fig 1: Map showing the study districts within Uganda. (Original shape files for the map were adopted by the authors with ArcGIS from the Ugandan Energy Sector GIS Working Group Open Data Site:* <http://data-energy-gis.opendata.arcgis.com/>)

**Comment 4**

Please review your reference list to ensure that it is complete and correct. If you have cited papers that have been retracted, please include the rationale for doing so in the manuscript text or remove these references and replace them with relevant current references. Any changes to the reference list should be mentioned in the rebuttal letter that accompanies your revised manuscript. If you need to cite a retracted article, indicate the article’s retracted status in the References list and also include a citation and full reference for the retraction notice.

*Response: Thank you for pointing this out. We have only added other references where needed but not removed as indicated below.*

| **Reference #** | **Reference** | **Lines** |
| --- | --- | --- |
| 47 | Nkamwesiga et al. (2023) | 150 |
| 48 | Nkamwesiga et al. (2020) | 150 |
| 49 | Nkamwesiga et al. (2019) | 150 |
| 50 | Ruget et al. (2019) | 150 |
| 51 | Tumusiime et al. (2019) | 150 |
| 52 | Tumusiime et al. (2023) | 150 |
| 53 | Nyakarahuka et al. (2016) | 151 |
| 58 | Akwongo et al. (2022) | 689 |
| 59 | Ruhweza et al. (2010) | 690 |
| 60 | Gitonga et al. (2016) | 690 |
| 62 | Nanfuka et al. (2018) | 701 |
| 66 | Ilukor et al. (2012) | 883 |

**Response to reviewers’ comments: Reviewer #1**

The reviewer appreciates the strong literature review provided and the efforts of the authors to detail the sampling process. The sites were selected on purpose based on epi reports in the areas. I suggest adding a section about the seroprevalence, vaccination coverage, and post-vaccination immunity levels for each disease and, if possible, per species (goats, sheep, cattle...) to make a case for the selections of those sites. Ideally, a table presenting those statistics nationally would easily justify the selection of sites on “purposes” unless I failed to understand the purposes mentioned.

*Response: We appreciate this valuable suggestion and thank you for pointing out the misunderstanding. The selection of the sites was based on epidemiology reports and livestock densities in those areas as highlighted in the paragraph starting from line 143 (Description of study sites), including references.*

Decision vs. Action/Operation is not just based on the household head. The literature on intrahousehold studies made several findings about who does what in the household regarding (decisions and actions) regarding husbandry, healthcare, markets/sales, and income spending. Drawing a list based on gender and household head might be a little too simplistic, given the objectives of the study. Some activities are not done by the women or men head of a household or rather by the young girl or boy in the household. It would have been more insightful to have them select activities and decisions they take and do in their household to ensure that the right person is in the room (FGD).

*Response: We agree with the reviewer’s assessment. Although the selection criteria for the FGD participants was based on the household head, the Focus Group Discussion tool was designed to tease out the gender roles for other household members such as girls/boys who could not be interviewed based on ethical issues. The assumption was that the selected adults would correctly give information about the roles/responsibilities of other household members, for example girl/boys. This information has been captured within the results as follows:*

*lines 327-328: The role of boys in ruminant disease management mostly in pastoral areas was limited to grazing, identifying unhealthy animals while out grazing and reporting to parents or guardians.*

*Lines 424426: Boys under the instruction of their fathers checked on grazing areas and watering points before taking the animals there.*

*lines 324-325: Girls supported their mothers to clean livestock shelters.*

*Lines 364-365: Both girls and boys handpicked ticks as part of the vector control measure.*

Provide a section of the SEM and show how the model has helped answer the research questions. The methods sections did not specifically explain how the SEM was built and used. Instead, all the results seem mainly based on FGD and KIIs, whose concrete findings are also barely mentioned. Hence, one can wonder if using FGD, KII, and participatory appraisals wouldn’t be enough to do such an analysis. Or what is the added value of the SEM?

*Response: Thank you for pointing this out. We have made the suggested changes, and these are highlighted in lines 169-178: The intrapersonal level explored attitudes, beliefs, perceptions and knowledge of PPR/RVF. The interpersonal level examined the social influences ranging from interactions with animal health workers, fellow livestock keepers, social functions in the community, family responsibilities and aspects of decision making. The community level explored veterinary/vaccination information outreach and feeding practices at community level, perception of livestock disease risk and outbreaks, while the institutional level considered vet service costs, distance to service centres, availability of animal health providers, information access and social economic inequalities between livestock keepers. The policy level explored rules and regulations regarding PPR/RVF prevention and control.*

One of the major animal health constraints in disease control is mobility data. In pastoral production systems, animals are rarely kept in fences, so mobility creates contacts at grazing, water points, and markets. The reviewer could not find real pieces of evidence in the articles on how these critical elements of disease control and prevention were handled.

*Response: This has been highlighted in lines 314-316 where in pastoral system-women identified and isolated sick animals in the morning before they are taken out for grazing.*

*Lines 424-426: Also, boys under the instruction of their fathers inspect water points before taking the animals for watering.*

*Lines 427-429: Goats and sheep which are identified as sick are tethered separately to avoid mixing them with the healthy ones or separated them in an isolated kraal until they are vaccinated or treated then released later for grazing.*

At the end of the participatory sections, were there any restitutions, lessons learned, or best practices for disease prevention and control to the participant? What have they learned differently from their initial knowledge? One of the results of the analysis was that women could have been misinformed or lacked access to the right information.

*Response: This is a great suggestion. The information generated from this study was used by partner organization mandated with veterinary extension service (VSF-G) to design awareness and sensitization campaigns for livestock keepers and veterinary extension workers on disease identification and preventive measures. Additionally, the key outcomes of this study have been summarized in a policy brief and shared with relevant authorities in the Ministry of Agriculture, Animal Industry and Fisheries (MAAIF) who are set to further incorporate it as part of their extension action point. Finally, the government developed a national PPR strategy document and even mobilized more than 1.5m doses of vaccines, not only due to this study but due to the evidence from the overall project.*

One aspect of disease prevention the reviewer expected was on herder’s willingness to vaccinate.  The reviewer appreciates the various elements in Fig 2, especially those on perceptions of vaccines (trust, availability, side effects) and WTV for vaccination. While the availability of vaccines and other drugs at the different shops is limited due to technical and infrastructural issues, it would have been an added value to inquire if money or the cost of vaccines represents a constraint for vaccination either during or outside of national campaigns.

*Response: The authors appreciate this suggestion. However, since this study was part of a wider project (Boosting Uganda’s investment in Livestock Development), other social economic aspects such as willingness to pay for PPR and RVF vaccines were considered in other social economic research studies on PPR and RVF which are in the pipeline for publication.* *However,* *RVF vaccine is not licensed for use in Uganda and PPR vaccines is not subsidized in Uganda. While the government procures the doses, the farmer must pay for the vaccine and the service (transport and administration through the government veterinarians). We have added some of these elements suggested to the conclusions (lines 761ff).*

The reviewer suggests adding a section for the conclusion as the authors did for the introduction. It is also important to make a clear differentiation between discussion and recommendation. Mixing the two has made that section overly long. Discuss only the study results first and add a couple of paragraphs in which you might provide your recommendations.

*Response: We appreciate this suggestion. We have added a section on conclusions and recommendations and a separate one on discussion.*

Lines 98: Reference with author name is provided in the text, unlike others. Kindly harmonize the references across the document. It may also apply to lines 643 and 634, though I am unsure. It is good to review this one more time throughout the document.

*Response: Thank you for highlighting this. We have reviewed the references across the entire document. Corrections have been made to lines 97,723, 753 and 774-777.*

Another point that stood out is the gender ratio for the KIIs. How sound is it for a gender-specific study that is trying to understand gender differences in disease control and prevention to interview 32 persons with only 6 women? This would have been statistically wrong. To some extent, by increasing the sample size, one would also expect to increase the chances for various experiences and knowledge shared of the subject.

*Response: Thank you for pointing this out. However, in our study this was not possible because finding female animal health professional in the study areas was challenging. Among all the animal health practitioners there were only 6 women. Given the low staffing level of women, we purposely targeted all these women who represent 100% of the female AHWs. The key informants revealed that even though vacancies were advertised at the district, very few women applied. This has been included under limitations.*

In Fig 2 and other parts (Table 2), where numbers are provided, it would be good to explain how those numbers are generated. You have multiple FGDs; each could be used to generate these tables or Figs. How did you end up with one table and a figure? Kindly expand the methods sections with an explanation of these questions.

*Response: The authors appreciate this suggestion. More information has been provided on how Fig 2 and Table 2 were generated.*

*Lines 235-246:* *To assess livestock disease prevention and control measures as well as the factors influencing disease control options across production system as reported by men and women (Fig 2), analytical themes were identified under each level of interest. The identified themes were grouped and clarified. The themes were then crosschecked by the authors and collaboratively revised. The control measures were categorized into within farm, between farm and community level. The influencing factors were analyzed along the social ecological themes by Mcleroy (intrapersonal, interpersonal, institutional, community and policy levels).*  *For further exploration, each theme was broken down into sub themes.* *For each sub theme, the number of mentions by each FGD, disaggregated by gender were counted and summed up across production systems. Results under each level of theme are presented in Table 2 and Fig 2 respectively.*

*Supporting information has also been provided for Fig 2 in S3* *Table.* *We have also made minor changes to Table 2 and figure 2 to improve readability.*

Your results are not presented per site or district but per animal production system… the reviewer is convinced that there isn’t a homogeneity of results across all sites. Hence, discussing the specificities of some of these sites could shed light on where or locations that need more attention and investments.

*Response: The author appreciates this comment and agrees with the suggestion. More information has been provided on the site-specific peculiarities in the results section in lines 307-673.*

**Response to reviewers’ comments: Reviewer #2**

The title of the paper is catch, and concise with the content of the paper. Abstract is relatively well written; it is with acceptable length and contains all mandatory information. The introduction subsection is well written; it gives an overview of the key issues contained in the paper. The theoretical orientation and analysis for the paper is well presented and they are relevant to the paper. The research questions are in line with the objectives of the paper, and they add clarity in the causal effect relationship of the key study variables.

*Response: Thank you very much.*

The methodology subsection is well written, the research design, sampling procedures and data analysis plans are relevant and adequate. However, despite the fact that the study was qualitative it is important also to acknowledge that quantitative data were also collected as it has been presented in Table 1 page 11

*Response: This is an excellent suggestion. We have indicated in the methodology that some quantitative data (demographic characteristics) was collected and analysed statistically (Lines 247-249) and (lines 284-287).*

There is a narrow use of the term gender to only refer to men and women, unless otherwise operationally defined, table 2 may require revisiting to include other gender categories e.g. youth male and female

*Response: We agree that this is a potential limitation of the study and we have highlighted this as a limitation (Lines 878-881).*

The presentation of the findings as it appears on page 13 -27 require revisiting by improving the citation of the qualitative data, the subsection should clearly show whether the findings are from FGD or KI, location and authority of the one giving such information in terms of the KIIs

*Response: The citations from the KII and FGDS as well as location have been added throughout the revised manuscript.*

The discussion of the findings requires some improvements, it should reduce the repetition of findings rather, synthesis it to generate conclusions in view of the key findings, the subsection also have minimal use of literature. After generating inferences from the finding the author should also show whether his/her finding compare with others and give justification why?

*Response: The discussion section has been updated, and as suggested the repetitions eliminated.*

The conclusions and recommendations are not glaring in the discussion subsection; it should be overhauled by either showing clear conclusion and recommendations within the same subsections or the new subsections on conclusions and recommendations be added

*Response: A new subsection on conclusions and recommendations has been added (Lines 797 ff.).*

*We are very grateful for the insightful comments and valuable improvements to our paper and thank the reviewers again for their time.*

*Best regards,*

*Jane Namatovu, Peter Lule, Marsy Asindu, Zoë A. Campbell, Dan Tumusiime, Henry Kiara, Bernard Bett, Kristina Roesel, Emily Ouma*
