# Supplementary material for: Gender roles in ruminant disease management in Uganda: Implications for the control of peste des petits ruminants and Rift Valley fever
Source: PLoS One. 2025 Apr 25;20(4):e0320991. doi: 10.1371/journal.pone.0320991 (PMC12027259; doi:10.1371/journal.pone.0320991)
Supplement: Attachment — Submitted filename: Response_to_Reviewers_auresp_2.docx [file pone.0320991.s007.docx]

Dr. Nussieba A. Osman

Academic Editor

PLOS ONE

September 26, 2024

**Re: Revision required for manuscript PONE-D-24-01412R1**

Dear Sir,

We appreciate your feedback and the opportunity to resubmit a revised manuscript. We also appreciate the reviewers for taking the time to carefully review the manuscript and give detailed and constructive comments. We have incorporated the changes reflecting all the suggestions provided by the editor and reviewers. For ease of reading, we have grouped our responses by section.

Below is our point-by-point response to each comment. If changes were made in the revised manuscript we indicate line numbers referring to the revised manuscript in its track changes version.

**Editors Comments**:

The manuscript needs major revision in order to be accepted for publications in PLOS ONE. Please revise your manuscript considering all points raised by the reviewers.

**Response**: Thank you for this valuable comment and suggestion, we have carefully revised the manuscript considering all the reviewers’ comments.

**Reviewers’ comments:**

**Reviewers: #1**

Let me start by thanking you and the rest of your team for the improved manuscript quality. Overall, you found my comments and questions insightful. I appreciate your positive feedback. I have recommended accepting the revised version of the manuscript. However, it would be best for you and your team to proofread the document for additional typos here and there.

**Response:** Thank you for your kind consideration and for the valuable comments. We have done our best to improve the manuscript regarding typos and grammar.

Besides, I suggest adding a line or two to account for the study's limitations and list all assumptions made. I still have some reservations that a household head who is usually more on the management and decision-making side would know enough about other tasks completed that are often overlooked by other members who are only sometimes underage (responding to your ethical comment).

**Response:** We have considered this as a limitation and reflected on it in lines 818-821.

The changes made to the suggested tables and figures and the additional sections for the conclusion and recommendations are all well-suited. The reviewer genuinely appreciates the upcoming extension efforts to raise awareness and prompt behavioral changes through a partnership with the veterinary extension service (VSF-G) and the Ministry of Agriculture, Animal Industry and Fisheries (MAAIF).

**Response:** Thank you very much.

**Reviewer #3**

I read the clear version of the revised manuscript appearing first in the pdf, line numbering refers to that version. The introduction introduces the subject, the local setting concerning PPR and RVF, the research questions and the methodologies to be used in a very nice way.

**Response:** Thank you very much.

**Abstract:**

Line 30: in the abstract you might want to skip mentioning the name of the software used.

**Response:** Thank you for the suggestion. This has been removed.

**Introduction:**

Line 46-49: the sentence is very long, please shorten it or divide it in two.

**Response:** The sentence has been divided into two, refer to Line 47.

Line 61-62: milking is an important activity that should be mentioned here.

**Response:** This is an important observation, thank you, and has been added; line 65.

Line 62: I presume the “in” should be placed after “children”.

**Response:** It has been moved; line 66.

Line 91: instead of “burning and burying” I would prefer “safe disposal of animals that have died from diseases” as there are several ways to do this without spreading disease

**Response:** This has been revised; Line 104.

Line 93: you need to mention the vaccination guidelines also for PPR.

**Response:** A national strategy was developed to guide the control and eradication of PPR in Uganda (31) which references different existing policies, such as the Animal Diseases Act, the Cattle Grazing Act and the Cattle Traders Act. Line 105.

Line 93: for RVF, safe handling of abortions (which might present without the animal being “sick)” is also a very important preventive measure, please add this

**Response:** This has been added. Please refer to line 119.

Line 95-107: this seems not to belong to the subsection PPR and RVF in Uganda but rather to the previous, please move and integrate to make sure to avoid overlap

Response: This has been moved and integrated under the gender and livestock section lines 72-77.

Line 108-132: This also seems not to belong to the subsection PPR and RVF in Uganda but rather to a subsection on gender in decision making and/or decision-making determinant models. Please move/integrate.

Response: This has been moved and integrated into the methods section; refer to lines 134-135

Line 116: which are these five key determinants? Are they only/exactly five? Please clarify.

Response: There are exactly five, including intrapersonal, interpersonal, institutional, and community factors. Please refer to lines 186.

Line 127: what is meant with “all-round” in this context?

**Response**: This was used to imply that the use of the SEM in livestock disease management allows for an examination of factors at multiple levels and provides a detailed view from individual behaviors to broader policy issues. We have revised this to make it clearer, see lines 140-142 in the methods section.

Line 128: more realistic than what?

**Response**: More realistic than without including livestock, which was previously rarely done. However, we have revised this in line 140-142.

Line 78: the abbreviation (SR) not previously introduced

**Response:** This has been corrected at this first mention; please refer to line 97.

Line 87: please remove “as an asset”, or explain why these words are needed here

**Response:** This has been removed, refer to line 124.

Line 89: please remove “chances of

**Response:** This has been removed. line 103

Line 133: this seems to be the beginning of another subsection? Please clarify. It would further be a good place to introduce the objectives of the study.

**Response:** This has been revised as per lines 127-129.

**Methodology**

Fig 1: I presume that the “corridor region” in the legend refers to the cattle corridor? I further don’t see any reference to the cattle corridor in the text (but I might have missed that), please remove the cattle corridor from the map or mention it in the description of the study area(s).

**Response:** The cattle corridor region has now been mentioned in the text, please refer to lines 151-154 and reference 49 (Nakiguli et al 2023).

Line 150: please define pastoral/agropastoral

**Response:** This has been defined. Please refer to lines 166-172 and reference 57 (Otte et al 2020).

Line 153: do you mean disease transmission or disease prevalence here?

**Response:** We have maintained ‘transmission’ implying that it is high, especially since wild animals are reservoirs to some livestock diseases (lines 173-174).

Line 191-193: the description of the GAHW and CAHW-workforce here seems misplaced, were CAHWs included as key-informants? Please clarify.

**Response:** The CAHWs were included as key informants since they supported the AHWs within the communities especially in very remote areas that are hard to reach. We have clarified this in lines 243-250.

The description of the methodology, especially data analysis, lacks important clarity and details, see the detailed comments below. Among the missing details not mentioned in the comments below is a section on the researchers’ positionality. As deductive coding is used it is important to reflect and mention the researchers’ previous knowledge and experiences to explain how the analysis process was driven by the researchers’

**Response:** Thank you for pointing this out. We highlighted the researchers’ positionality in the limitations section at the end of the discussion, please refer to lines 823-829 and reference 68 (Gary A, 2020).

It is further not clear from either the methodology or the results how the SEM and the GAF was used, it now appears that the SEM was only use was to divide the factors influencing disease control decision in the five mentioned categories and there is no description of GAF-analysis in the methodology at all.

**Response:** The GAF analysis has been added to the methodology section, lines 134-137

Moreover, it is not clear how the deductive codes described in the code books (S1 and S2) was used in the analysis to give the subsections described in the results, or if these subsections are indeed what is described as themes/common themes/analytical themes. This lack in the description of the methodology and the analysis makes it impossible to judge the methodological rigour, and if the conclusions are based on the data.

Line 221-232: please clarify the analysis- process. Did you really code before uploading the data in Nvivo? Did you use deductive coding or were themes emerging from the data? It seems (but this is not clearly described) that deductive coding was used, with the codes used in the respective code book for RVF and PPR), this needs to be described in more details, and in my opinion the codes should be included in the manuscript (not as supplementary material).

**Response:** Thank you for pointing this out and for the suggestion. We have added additional detail about how these codes were used within the manuscript. Please refer to lines 268-274. The codebooks are still provided in the supplementary materials for readers who prefer the table format.

Line 228: please clarify what you mean by “assess” in this regard (number of, presumed efficiency, quality, cost-benefit etc).

**Response:** We have reworded to be clearer about the data analysis process; Lines 273-274.

Line 255-258: this belongs in the methods-section. In addition, it is stated here that the factors were organized according to the SEM, which is what I commented on in the general comment, did the SEM include some more analytical elements, or only to organize the factors according to these pre-defined categories?

**Response:** We apologize for any lack of clarity in the methodology on the raised points. We have tried to be clearer in the methods section now; please refer to lines 289-290.

After reviewing and familiarizing with the transcripts, an initial coding frame was developed, drawing on both the transcript data, the GAF and SEM. The gender activities, resources/services that men and women access to prevent or control disease were identified. The initial codes included: gendered livestock ownership, gendered roles as well as preventive and control measures. The preventive and control measures were sub-coded between men’s and women’s measures (treatment, vector control, culling, movement control and vaccination). All coded data for PPR and RVF data sets were read and re-read to identify connections, similarities or differences between codes. Based on emerging patterns and relationships, codes were organized into overarching themes disaggregated by gender and production system. Unique to RVF were the risky practices/behaviors predisposing animals/humans to zoonoses, but majority of the codes were overlapping PPR and RVF data sets. Thereafter, themes were generated. The themes and the coded data were reviewed to ensure accuracy in the captured data each theme. The measures were then categorized into within farm, between farm and community level depending on where the activity took place. A similar analysis was conducted on the influencing factors, which involved identifying all the factors influencing each choice of a disease preventive and control method. This analysis focused on the underlying factors which determined the gender roles, access to and control over resources/services. These included source of services/information, availability/accessibility/affordability of services, perceptions/views/concerns towards a method, knowledge/awareness, socio-cultural factors, decision-making aspects, trust and experience with a method. The aim of the analysis was to identify and contrast sources of services/resources and to understand their perception/concerns and constraints regarding a particular preventive and control measure. The identified themes were then categorized and analyzed along the social ecological themes by Mcleroy (intrapersonal, interpersonal, institutional, community and policy levels).

I have further have some major concerns about the FGDs, see my comment on S1 below.

Supplementary material 1 (FGD guide): The FGD-guide includes very many and quite detailed questions, even if only considering section A. With section B and C the content becomes enormous. It is written in the S1-file that different FGDs should be used for section A versus sections B and C, but this is not reflected in the manuscript, was those sections used for another study? Please clarify. I also find it unusual to have so many detailed questions in a FGD in which one normally aims at having a free and open discussion driven by the participants. With so many questions the guide looks more like a semi-structured protocol for an individual interview. The length/content of the guide further raises several questions:

Line 205: according to the FGD-guide in S1 the PE-tools were not a complement, but an integrated part of the FGDs? Please clarify and report of the results from the PE-exercises.

How long time did the FGDs take? How was participant engagement ensured during the entire process? How was the results used, as for example the results from the PE are not included in this manuscript?

For question 3a the probing questions and the instruction to the facilitator don’t seem to match (common diseases/all diseases/description of the diseases/zoonotic potential vs ranking based on highest mortality). Were the follow up questions (which are very many) in questions 4-6 asked for all listed diseases? If not, which ones and how was the selection agreed on?

**Response:** We appreciate the reviewer for all the comments highlighted on the FGD and KII guide. We acknowledge that this was a challenge as each FGD took half a day, but the participants were facilitated with coffee/lunch breaks

We also agree with the comments on the bulkiness of the KII guide, but the questions included questions for all categories of KIIs, e.g. CAHW, AHO, Policy makers etc.

With regards to the other results from the FGDs which are outside the scope of this paper, the information generated through this study was used to write two research briefs one under PPR and another one under RVF. In the attached FGD tool, we have italicized those questions that were used for other outputs

Below are the links to the different project outputs from the FGD findings.

PPR brief: <https://hdl.handle.net/10568/118339>

RVF brief: <https://hdl.handle.net/10568/115766>

RVF poster: <https://hdl.handle.net/10568/115648>

PPR posters: <https://hdl.handle.net/10568/115639>; https://hdl.handle.net/10568/124997

Some of the results from the PE especially the one from the resource mapping was used to generate information on resource use (grazing, feeding, marketing and veterinary services) and availability of these resources to both men and women within these communities. Part of this information was used to write a research brief whereas the remaining information was used to build on some of the explanations under the factors influencing disease prevention.

Line 207: you need to add some more information on facilitation, language and translation. Who in the research team facilitated the interviews (FGDs and KIIs)? In what language(s) was the FGDs and KIIs done? By whom in the research team?

**Response:** Four of the co-authors (Ugandans) facilitated the KIIs in English. Some of the KII were done over the telephone due to COVID restrictions in some of the study regions at the time.

For the FGDs –local facilitators interfaced with the participants and were supervised by one of the Ugandan co-authors. Using the Group Discussion Guide, the trained local facilitators led the FGDs in the local language of each district while the note takers took the notes verbatim. Men facilitated men groups while women facilitated women groups. The FGDs were done in the local languages as described in lines (255-257). We have made changes considering this comment.

By whom in the research team? Was notes taken in English? If not – were the notes translated?

Response: The KII notes were taken in English while the FGD notes were taken in the local language and translated in English by a designated note taker for each district who was fluent in the language of the participants and familiar with their culture. Line 259.

How/by whom in the research team translated the interview guides? The local facilitators from respective district

Response: The designated note takers and facilitators from each district were taken through the question guides and associated probes in a two-day workshop to ensure that they understood the purpose and intent of each question before interpreting them. A second interpreter was engaged to translate the guides back to English to avoid any potential judgmental language or interpretation bias.

What do you mean by “in person”?

**Response:** Some of the KII were done over the telephone due to COVID restrictions in some of the regions at the time. (Line 252). For the FGDs – the local facilitators interfaced with the participants and were supervised by a Ugandan co-author.

Line 219: are the “team members” co-authours? Please clarify.

**Response:** By ‘team member’ we meant the ‘local facilitators’ who transcribed verbatim and translated the audio recordings into English. This has been revised (line 259).

Line 223: please mention the research questions.

**Response:** These have been mentioned in lines 127-133.

Line 224-231: please clarify the difference between/define what you mean by: theme /analytical theme /common theme /identified theme –

**Response:** Thank you for highlighting this. We have harmonized these terms to refer to themes. We had used these terms interchangeably and these referred to response patterns or descriptions that respondents gave regarding a particular topic for example (treatment methods). These were identified, grouped together and analyzed/inferenced by the research question.

**Results**

Line 254: see my comments on the analysis and the mentioning of different kinds of themes. It would be good to start the results section by mentioning the themes that arose (emerged) during the analysis (if any).

**Response:** Thank you for this suggestion. The themes that arose have been introduced in the result section. Please refer to lines 315-318.

We present the results of the thematic analysis in two sections: prevention and control measures for ruminant disease (within farm, between farm, and at the community level) and factors influencing disease prevention and control (intrapersonal, interpersonal, institutional, community-level, and policy-level).

Line 255-258: this belongs in the methods-section. In addition, it is stated here that the factors were organized according to the SEM, which is what I commented on in the general comment, did the SEM include some more analytical elements, or only to organize the factors according to these pre-defined categories?

**Response:** Yes, the SEM included some analytical elements, and these have been explained within the manuscript; please refer to lines 318-334.

Line 255-258: this belongs in the methods-section

Response: Thank you; this has been moved and integrated to the methods section (line 265).

Line 256: I presume the “socio-ecological framework” is the same as the SEM? Please clarify

**Response:** We apologize for the mix-up of the terminologies. We meant the SEM, and this has been harmonized within the manuscript.

Table 1 and 2: please adjust the formatting. Regarding the note for Table 1: I don’t see any SDs in this table? Footnotes are normally used for this kind of information.

**Response:** The demographic data for the focus group discussants is not meant to be generalized to a larger population given they were not randomly selected, therefore we haven’t provided additional statistics such as standard deviation. We suggest the demographic data be used to get a sense of the respondent characteristics only.

Table 2 and Figure 2: does “mixed” refer to “mixed crop-livestock system” or a mix of pastoral/agropastoral systems? Please clarify.

Response: ‘Mixed” referred to “mixed crop-livestock system”. This has been clearly defined. Line 52.

Line 282: please check the level of the subsection titles to make the reading easier and clearer.

**Response:** The subsection titles prevention and control of ruminant diseases have been reduced to 3 levels only (prevention and control measures within farm, preventive and control measures between farms, preventive and control measures at community level (lines 345-464).

All through the results section it needs to be made clearer if the reported results are valid for all systems, a specific system, or only one specific district.

Line 297: is this statement valid for all agropastoral study areas or only for Isingiro? Please clarify.

**Response:** This was only valid for Isingiro, and it has been clarified; line 515.

Line 302-317: this seems not to fit under prophylactic measures, please move to another subsection.

**Response:** Moved and integrated (lines 503-505).

Line 322: the purpose of this statement is not clear. Was spraying done more frequently (compared to pastoral or mixed systems?) in agropastoral systems, or was it done more as a family activity (compared to what/where?). Please clarify.

**Response:** This has been clarified within the manuscript. Please refer to lines 365-367.

should it be” kids and lambs” or were only goats kept. Please clarify.

**Response:** Revised to both kids and lambs (Line 372-373).

Line 303: was this only done in “sudden deaths”? Or do you mean in all cases of animals dying (by themselves) without known cause of death?

**Response:** This was done is case of unknown diseases leading to sudden death. It has been clarified within the manuscript. Lines 392-394

Line 303-304: please consider the wording “local postmortem”, I think maybe something like “carcass opening” would be a more correct description

**Response**: This has been revised (line 392-394).

Line 305-307: was the meat distributed and the skin used as described only if the animal had died a “sudden death”? Please clarify.

**Response:** Yes, this was so because animals were rarely slaughtered for home consumption as illustrated below:

*My husband does not buy meat for us and does not want to slaughter for home consumption, yet it’s my children and I who graze, so it will be an advantage to have meat when the animal dies.* (Women FGD, Nakapiripirit, Pastoral system)

Line 308-09 and 312-14: these 2 quotes seem to illustrate the same thing, please remove one

**Response:** We felt that the inclusion of these 3 quotes would demonstrate how the issue was experienced in different production systems.

In the revised manuscript, we have maintained only one quote. Line 508-511

Line 308: quotes are normally written in italics.

**Response**: The quotes have been revised accordingly throughout the results to appear in quotations within the text if less than one line or italicized without quotation marks and indented if longer than one line.

Line 365: the mentioning “from the FGDs” are puzzling as it seemed to me that all results reported so far were from the FGDs (compare line 256). Please clarify.

**Response**: This has been clarified (line 424).

Line 368-369: please remove “on the farm”.

**Response:** This has been deleted (line 426).

Line 399-403: this sentence seems to be related to the one on line 385? Please put them together and avoid repeating/overlapping

**Response:** This has been moved and integrated (lines 446-450).

Line 385: what is a “personal farm”

**Response:** This has been revised to “individually owned farms” (line 448)

and what do you mean by “shifting” in this sentence? Please clarify.

**Response:** This has been revised to “relocating” (line 449).

Line 387: how was movement limited by women? Please clarify.

**Response:** This has been clarified in line 450 as indicated below.

Women avoided mixing their animals with the neighbors by tethering them while grazing, and feeding them from home, and providing drinking water in separate water troughs.

Line 392: at what occasion were animals moved? Please clarify.

**Response:** This has been clarified from line 454 as indicated below.

In the case of individually owned kraals, healthy animals were separated from the sick ones, during a disease outbreak moved to a different location on the same land, and continuously monitored.

Line 443: it needs to be described in the methodology section how this order of importance was established. Please clarify.

**Response:** Revised in lines 500-502

Line 485: please introduce the quote with a full sentence.

**Response:** Revised from line 542.

**Discussion**

The discussion lacks depth, and the discussion of the findings is not on the level of abstraction and reflection that is expected from a qualitative study in this journal.

**Response:** Thank you for pointing this out. We have further reflected on findings and tried to align the findings presented throughout the paper, for example in the abstract (lines 31-40) and discussion (Lines 704-709).

Line 655-656: I failed to see description of the gender analysis framework in methodology section, or the results coming from this method. Please clarify.

**Response:** The gender analysis framework has been described in the methodology (135-137) Examples of results coming from this method include lines 711-716 (livestock management roles and disease identification) and access to resources for women (774-776).

Line 659-661: this seems to be results that are not reported in the results-section. Please don’t introduce new results in the discussion section.

**Response:** This has been revised accordingly. Please refer to lines 1180-1182

Line 661-663: I don’t understand this sentence, please try to clarify/rewrite.

**Response:** This has been revised, then moved and integrated in from line 822.

Line 663-666: idem. Plus the study does not seem to be designed to assess risk-factors so I don’t understand how the statement is based on the results?

**Response:** This has been deleted.

Line 667: please clarify what “more” refers to (more than what)?

**Response:** This was meant to explain that within farm measures were more implemented than measures between farm and at community level.

**Response:** This sentence has been clarified Line 722-725

Line 678 and in other places: I think “self-medication” is not the best term for what you want to describe, for me “self-medication” sounds like someone is medicating him/herself. Please look for another way to describe the phenomenon of medicating one’s animals without consulting an animal health professional

**Response:** This language has been removed throughout and replaced, e.g. line 730.

Reports of livestock keepers purchasing veterinary drugs and acaracides to administer to their livestock themselves suggests an ease of access.

Line 698-670: I don’t understand this sentence, please try to clarify/rewrite.

**Response:** This sentence has removed (Line 700) as quarantine policy does not directly relate to RVF or PPR control in Uganda.

Line 698: I presume you mean “more common”? Please check.

**Response:** This sentence has been revised. Line 738 onwards.

Line 690-692: I confess to not having read the article in the reference, but disregarding what it says, the association between purchasing acaricides and domestic violence and in the next step suicide needs to be more thoroughly introduced here.

Response: This has moved and better explained in the results section, lines 379-382

In some areas, cost is not the only restriction women face accessing veterinary products. Reports on suicidal tendencies among women in pastoral areas of Uganda resulting from domestic violence and spousal disputes have led to restrictions on the sale of acaricides and related chemicals in some veterinary drug shops to women (21).

Line 781-798: this seems not to be conclusions or recommendations, please move to a more appropriate section.

**Response:** This has been moved to line 759 in the discussion.

Line 782: I presume you mean “reported occurrence”?

**Response:** This has been revised in line 809.

**Editorial concerns**

The text would further highly benefit from professional language editing.

**Response:** One of our co-authors (ZC) has done her best to edit better.

Line 144-147: a blank is missing or is in surplus in a couple of places in this sentence, please check and correct.

**Response:** This has been addressed in multiple places.

Line 268-69: a verb seems to be missing in the first part of the sentence.

**Response:** A verb has been added in line 412 and whole paragraph revised (lines 413-417).

Line 294-295: please check the sentence for grammar and syntax

**Response:** This has been revised (lines 439-445).

Line 295-296: please check the sentence for grammar and synthax

**Response:** This has been revised (lines 439-445).

Line 377, 381, 396: please check for typos.

Line 377, 381, 396: please check for typos.

Line 410-413: please check the sentence for grammar and syntax.

Line 507-509: please check the sentence for grammar and syntax.

Line 669; a blank is missing before the bracket.

Line 689-690: please check the sentence for syntax.

Line 726-728: please check the sentence for syntax.

Line 694-696: please check the sentence for grammar and syntax

**Response:** All the above comments on grammar, syntax, and typos have been revised. In addition, we have made other minor edits to flow, grammar, and syntax to make the manuscript easier to read without changing the original content.

We look forward to hearing from you and to responding to any further questions and comments you may have.

Sincerely

Jane Namatovu, Peter Lule, Marsy Asindu, Zoë A. Campbell, Dan Tumusiime, Henry Kiara, Bernard Bett, Kristina Roesel, Emily Ouma
