# Supplementary material for: Gender roles in ruminant disease management in Uganda: Implications for the control of peste des petits ruminants and Rift Valley fever
Source: PLoS One. 2025 Apr 25;20(4):e0320991. doi: 10.1371/journal.pone.0320991 (PMC12027259; doi:10.1371/journal.pone.0320991)
Supplement: Attachment — Submitted filename: Response_to_Reviewers_auresp_4.docx [file pone.0320991.s009.docx]

Dr. Nussieba A. Osman

Academic Editor

PLOS ONE

December 25, 2024

**Re: Revision required for manuscript PONE-D-24-01412R3**

Dear Dr. Osman,

We would like to thank the academic editor and the reviewer once again for taking the time to review our manuscript and for the relevant remarks and comments which have helped us to improve the quality of our paper. We have done a careful re-reading of our paper and addressed the comments raised. We have also corrected some typographical errors.

These changes are detailed below.

**Editor’s Comments**:

Please review your reference list to ensure that it is complete and correct. If you have cited papers that have been retracted, please include the rationale for doing so in the manuscript text or remove these references and replace them with relevant current references.

Any changes to the reference list should be mentioned in the rebuttal letter that accompanies your revised manuscript. If you need to cite a retracted article, indicate the article’s retracted status in the References list and also include a citation and full reference for the retraction notice.

**Response**: Thanks for these valuable comments. We have rechecked the reference list thoroughly and found one reference (ref 10) Dudi et al (2019) which we have removed as it is no longer available online.

**Reviewer’s comments:**

**Introduction**

Line 59: isn’t “vaccination and breeding” part of animal husbandry? Maybe you mean “daily care” or something similar?

Response: Thank you for highlighting this. We have revised manuscript; The roles of women have been broken down to feeding animals, fodder collection, and cleaning animal sheds. Lines 58-60.

Line 89: does this statement only refer to one RVF-vaccine (“the RVF vaccine” (and which in that case), or should it be” RVF-vaccines”?

Response: This has been revised to read “vaccines” as suggested. Line 92.

Line 124:as an investment or savings” seems redundant here, please remove.

Response: We have maintained investment: Please refer to Line 128.

**Methodology**

Many of my questions and comments have been answered but not all. Some has been answered in the point-to-point-answers to me, but with no changes done to the text.

Generally, if a question is posed in the reviewer report, it implies that changes are needed in the text, it will seldom be sufficient to just provide an answer to the reviewer.
Questions from previous review that need to be answered or incorporated in the text and not only answered in the point-to-point answers:

Response: We appreciate your specifications of the pending questions posed in the previous review and not indicated in the text. We have made changes considering the comments highlighted.

How/by whom in the research team translated the interview guides? In what language(s) was the FGDs and KIIs done? How long time did the FGDs take?

Response: We have incorporated the changes below in the new version of the manuscript.

Lines 226-231: Prior to conducting the research activities, a team of four people fluent in the respective local languages (two elders, two animal health practitioners from each study district) translated the study guide. The translated guide was further refined by the local facilitators from each district two-day training workshop. The enumerators were taken through the questions and associated probes to ensure that they understood the purpose and intent of each question.

Regarding who facilitated the interviews (FGDs and KIIs) in the research team:

The trained local facilitators led the FGDs in the local language of each district while the note takers took the notes verbatim. Men facilitated men’s groups while women facilitated women’s groups. The local facilitators and note takers were supervised by one of the Ugandan co-authors. Using the FGD guide, four of the co-authors (Ugandans) facilitated the KIIs in English. Some of the KII were done over the telephone due to COVID restrictions in some of the study regions at the time.

For the FGDs, local facilitators interfaced with the participants and were supervised by one of the Ugandan co-authors. Using the Group Discussion Guide, the trained local facilitators led the FGDs in the local language of each district while the note takers took the notes verbatim.

The FGDs were done in the local languages as described in lines (281-283):

Karamojong for Napak and Nakapiripirit, Runyankole for Isingiro, Luganda for Sembabule, Ateso for Serere, and Luganda, Ateso, or Lugweere for Butebo.

According to the FGD-guide in S1 the PE-tools were not a complement, but an integrated part of the FGDs? Please clarify and report of the results from the PE-exercises.

Response: We agree with the reviewer’s comment. The PE tools were part of the FGDs. Some of the results from the PE, especially resource mapping, were used to generate information on resource use (grazing, feeding, marketing and veterinary services) and availability of these resources to both men and women within these communities. Since the FGD tool covered several aspects and served several purposes, part of this information was used to build on some of the explanations under the factors influencing disease prevention. The PE results were also used to write research briefs and non-technical policy briefs for the Ministry of Agriculture, Animal Industries and Fisheries in Uganda.

How was the results used, for example the results from the PE are not included in this manuscript?

Response: With regards to the other results from the FGDs which are outside the scope of this paper, the information generated through this study was used to write two research briefs, one under PPR and another one under RVF, posters and blogs as indicated below; Please refer to lines 258-259.

| Reference number | Reference |
| --- | --- |
| 62 | Asindu et al (2021) |
| 63 | Asindu et al (2021) |
| 64 | Lule et al (2022) |
| 65 | Lule et al (2021) |
| 66 | Namatovu et al (2022) |

In the attached FGD tool, we have italicized those questions that were used for other outputs.

How was participant engagement ensured during the entire process?

Response: Various participatory tools were used to facilitate discussions and ensure participant engagement. These included simple ranking and proportional piling. Please refer to lines 241-244 (references 60 and 61): Energizers and coffee/lunch breaks were provided in between sessions.

For question 3a the probing questions and the instruction to the facilitator don’t seem to match (common diseases/all diseases/description of the diseases/zoonotic potential vs ranking based on highest mortality). Were the follow up questions (which are very many) in questions 4-6 asked for all listed diseases? If not, which ones and how was the selection agreed on?

Response: We appreciate your constructive critique, which helps us to clarify more. This was an error that was corrected. Refer to S1 File

As regards whether the follow-up questions were asked, these were asked with the intent of exploring awareness of livestock keepers on livestock diseases affecting their SR/Cattle including PPR and RVF. Please refer to lines 235-240 and 253-254.

First, participants listed the common disease problems that affected their goats/sheep/cattle and described the signs of each disease (local disease names were noted). For the ranking of diseases, 100 beans/counters were distributed among the diseases to indicate their relative importance. In the process, participants were asked to explain the reasons for assigning the scores. The participants were asked to identify the causes of the diseases mentioned, which diseases caused more deaths, and which age groups were most affected. Participants were then asked to explain how each disease was spreading between animals and whether it could be transmitted between animals and people, and who of the household was involved in activities that might lead to disease transmission. Ways of transmission were listed (within farm, between farm, community level). Participants were asked to explain what they did in response to the mentioned diseases to understand their disease coping strategies

Line 136 and 138: please use the abbreviations (GAF and SEM) all through once introduced.

Response: This has been revised, the acronyms are introduced in lines 138-140 then used throughout the manuscript.

Line 136: do you mean “elders” or “the elderly”? Please clarify.

Response: This has been revised to elderly. Please refer to line 141.

Line 260: were the facilitators part of the research team?

Yes, they were part of the research team. Please refer to line 287-288.

Line 291: Is the “socio-ecological themes” the same as the SEM?

Response: Yes, this has been revised to SEM.

Line 290 and 202: are “identified themes” and “selected themed” the same?

Response: This has been revised to selected theme to imply that predetermined themes were applied to the factors influencing livestock disease prevention and control. Please refer to line 317

Lines 316-319: it seems like the GAF (or part thereof, see below) was applied to the first section and the SEM to the second section? Was this the case? If so, please clarify this in the M&M and results.

Response: This has been clarified in the Methods section, lines 293-294.

Lines 322-325: are these results? If so please check the tempus used, if not, move to introduction.

Response: Yes, these are results. We have revised the sentence to make it clearer. (lines 347-351).

Lines 329-331: I don’t understand what you mean with this sentence, isn’t that what you will do using the GAF and SEM?

Response: Yes, this is what is done with the GAF and SEM. The sentence has been deleted.

Line 345-387: this section seems to analyse/describe the different measures and tasks performed by men and women and to some extent boys and girls. If this is meant to represent the GAF-analysis, you need to mention somewhere that you analyse according to GAF – minus the elderly.

Response: Thank you for the suggestion. This has been added in line 342-343.

Lines 380-382: see my comment on this in the previous report:

**Comment from the previous review**: I confess to not having read the article in reference, but disregarding what it says, the association between purchasing acaricides and domestic violence and in the next step suicide needs to be more thoroughly introduced here:

Response: We have thoroughly explained the connection between acaricide use, domestic violence, and suicidal tendences among women in Karamoja as follows.

Lines 88-91: Given the high incidences of domestic violence against women in Karamoja (22), and cases of women using acaricides to poison themselves to escape from domestic abuse, women have been banned from purchasing acaricides (20).

Lines 403-405: Some veterinary drug shops in the pastoral areas of Karamoja have banned the selling of acaricides and associated chemicals to women due to reports of suicidal thoughts among women in these areas as a result of domestic abuse and marital conflicts (20).

Line 475: Please change to “foot and mouth disease”.

Response: Revised accordingly. Line 499

Line 680: I think “vaccine” should be “vaccinate”?

Response: Thanks very much for the suggestion. This has been revised to vaccinate.

Line 705-708: Isn’t that what you described in your results? Here in the discussion you are meant to discuss these results against literature.

Response: Thanks very much for highlighting this. The whole sentence has been deleted.

Line 717: suggestion: “might be limited for all categories (men, women, boys and girls) of participants”. Or did you see any gender differences in this regard?

Response: The gender specific difference has been captured within the text. Please refer to line 737, reference 66, Namatovu et al (2022): reference 69, Namatovu et al (2021)

Line 782: And themselves!

Response: Thank you for the suggestion. This has been added.

Lines 782-784: Maybe worth mentioning costs of transport and women’s restricted access to monetary resources here if you think it is relevant based on your results.

Response: Thanks very much for this valuable suggestion. We have expanded on the explanation within the text and taken into consideration women’s restricted monetary resources which might prevent them from meeting related transport costs (Lines 803-806

Lines 789-795: Maybe worth mentioning here that women faced specific challenge in accessing vaccination, not only because of being smallholders, but specifically related to gender? That is at least how I interpreted you results on this?

Response: As per the suggestion, we have mentioned the gender specific challenges within the text. Please refer to lines 802-809

Line 832 and forward: this section is length and mostly contains policy recommendations.

For clarity, I would recommend dividing it into (scientific) conclusions of the study, and policy recommendations regarding vaccination and disease control in Uganda (and similar contexts).

Response: We highly appreciate your guidance on this. We have categorized the recommendations and conclusions into policy and scientific conclusions.

Table 1: Please check the formatting.

Response: This has been formatted

**Typographical errors**

We have corrected the typographical errors and made some improvements to obtain a more readable version as indicated below.

Line 32: there seems to be an abundant “the” in this sentence.

Response: This has been deleted.

Line 77-81: please check this sentence for grammar/syntax.

Response: the whole sentence has been revised.

Line 48: the sentence is long with several sub-clauses and contains two “especially”, please rephrase.

Response: The sentence has been revised.

Line 82: this sentence seems to discuss the same theme as the previous so I think the blank line should be omitted.

Response: Thank you. This has been revised

Line 61: this sentence seems to discuss the same theme as the previous so I think the blank line should be omitted.

Thanks very much for these valuable suggestions, the blank line has been deleted.

Line 209: a “selected” seems to be missing.

Response: Thank you for highlighting this. The word ‘selected’ has been added. Please refer to line 212

Line 291: Please remove the redundant full stop.

Response: This has been deleted.

Line 105-107: please check this sentence for grammar/synthax.

Response: The sentence has been revised. Please refer to lines 109-111

Line 703: There seems to be a “what” missing after “asked”.

Response: Thank you, this has been added and the whole sentence revised

Sincerely,

Jane Namatovu, Peter Lule, Marsy Asindu, Zoë A. Campbell, Dan Tumusiime, Henry Kiara, Bernard Bett, Kristina Roesel, Emily Ouma
