# Supplementary material for: Gender roles in ruminant disease management in Uganda: Implications for the control of peste des petits ruminants and Rift Valley fever
Source: PLoS One. 2025 Apr 25;20(4):e0320991. doi: 10.1371/journal.pone.0320991 (PMC12027259; doi:10.1371/journal.pone.0320991)
Supplement: Attachment — Submitted filename: Response_to_Reviewers_auresp_5.docx [file pone.0320991.s010.docx]

Dr. Nussieba A. Osman

Academic Editor

PLOS ONE

January 27, 2025

**RE: Revision for Manuscript PONE-D-24-01412R4**

Dear Dr. Osman,

Thank you for giving us an opportunity to revise our manuscript for publication. We appreciate the time you and the reviewer have spent contributing feedback for us to strengthen our paper. We have made the following amendments to reflect the comments and suggestions.

**Editor Comments:**

**Comment:** Please review your reference list to ensure that it is complete and correct. If you have cited papers that have been retracted, please include the rationale for doing so in the manuscript text or remove these references and replace them with relevant current references. Any changes to the reference list should be mentioned in the rebuttal letter that accompanies your revised manuscript. If you need to cite a retracted article, indicate the article’s retracted status in the References list and also include a citation and full reference for the retraction notice.

Response: Thank you for the comment. We have checked the reference list and updated the accession numbers for the following references

| **Reference** | **Reference number** |
| --- | --- |
| Flintan, F (2008) | 15 |
| The Cross Cultural Foundations of Uganda (2020) | 22 |
| Nkamwesiga, J. et al (2022) | 26 |
| WOAH (2018) | 35 |
| Baudin, M. et al (2016) | 37 |
| Doran, K. et al (2017) | 45 |
| Sadarangani, TR. (2020) | 47 |
| March, C. et al (1999) | 48 |
| Nkamwesiga, J. et al (2020) | 52 |
| Nkamwesiga, J. et al. (2019) | 53 |
| Tumusiime, D. et al (2019) | 55 |
| Alemu et al (2019) | 60 |
| BOU (2021) | 68 |
| Dumas, SE (2018) | 69 |

**Reviewer #3 comments:**

**Comment:** The manuscript has improved a lot since the last revision, some minor, mainly editorial comments remain/have arisen due to changes in the text, see details with line numbers below.

Response: Thank you very much.

**Comment:** Apart from the minor comments I have a major concern with the discussion. It still lacks engagement with literature, and a large part of it is policy, veterinary extension and scientific recommendations. The latter is obviously an important output from research, but as this is a scientific contribution the equilibrium between recommendations and scientific discussion needs to better balanced. As the study has already produced other outputs (including recommendations), maybe the recommendations can be reduced here? Or included in another form (as a scientific discussion). As an example of the lack of engagement with the literature large parts of the discussion discuss the results, but without referring to the literature, e.g lines 748-774, 778-798, 811-820, 823-830.

Response: We appreciate your direction. We have revised the discussion and tried to engage more with existing gender and veterinary literature by incorporating the following references.

| **Reference** | **Reference number** | **Line number** |
| --- | --- | --- |
| United States Department of Agriculture (2012) | 75 | 751 |
| Dione, M. (2021) | 76 | 757 |
| Bishop, RP. et al (2023) | 77 | 760 |
| Nuvey, FS. et al (2022) | 78 | 762 |
| Serra, R. et al (2022) | 79 | 765 |
| Shibata, R. et al (2020) | 80 | 765 |
| Mburu, CM. et al (2023) | 81 | 777 |
| Robi, DT. et al (2023) | 82 | 779 |
| Williams, S. et al (2022) | 83 | 780 |
| Ahmed, H. et al (2021) | 84 | 782 |
| Bjornlund, H. et al (2019) | 86 | 791 |
| Krause, BL (2024) | 87 | 793 |
| Malapit, H. et al (2020) | 88 | 793 |
| Po, JYT et al (2020) | 89 | 793 |
| Mutua, E. et al (2017) | 90 | 806 |
| Kiara, H. et al (2017) | 93 | 833 |
| Conrad, B. et al (2024) | 94 | 835 |
| IDRC (2023) | 96 | 888 |

**Comment:** Line 219-225: Please state in which district the “extra” 4 FGD was held, and why (6 districts*2 subcounties in each*2FGD in each soubcounty (one men and one women) =24, but it seems like 28 FGDs were held?).

**Response:** Thank you so much for highlighting this. In short, the PPR sites covered three districts and the RVF sites covered four districts (lines 163-165) , which led to 12 and 16 FGDs respectively.

We added some text to clarify:

Lines 210-214

“In total, 28 FGDs were conducted: 16 FGDs with women and men keeping cattle in the RVF districts, and 12 FGDs with women and men keeping small ruminants in the PPR sites. The numbers were slightly different because the RVF sites had four enrolled districts and the PPR sites had only three, with Isingiro District selected for both diseases.”

**Comment:** Line 232-233: It seems like the sentence starting on line 232 belongs to the previous sub-section, and that the sentence starting on line 233 belongs to a new subsection, i.e. that a blank line should be introduced between these two sentences? Please revise.

Response: This has been revised. Please refer to lines 234 and 237.

**Comment:** Line 250: What do you mean with “various aspects of the FGD-guide” in this context? Do you mean “since the FGD-guide covered many topics” or something similar? Please revise.

Response: Thank you for pointing this out. This has been revised to “Since the FGD guide covered many topics”, Line 254

**Comment:** Line 469: This is confusing, restricting access to neighbours’ animals/movement seems to imply a belief that disease are spread by direct contact and not by ticks? Pease clarify.

**Response**: We apologize for the lack of clarity. We have revised the sentence to make it clearer: Line 472

“Men in the mixed and agropastoral systems believed that direct contact led to spread of tick-borne diseases to their livestock, and as a result, took action to restrict animal movement to avoid contact with livestock from neighboring farms.”

**Comment:** Line 473-474: Do you mean in both areas? Pease clarify.

**Response:** This has been revised to indicate the difference in practices in the two areas. Line 477-478

“Fencing individually owned farms at household level in agropastoral and relocating animal kraals in pastoral areas were done more for cattle than for SRs.”

**Comment:** Line 499/502: Is the different use of the abbreviation/disease name in full in these two quotes a mirror of how the discussants used terms, or an oversight from the authors? Please check this up.

**Response:** Thanks for pointing this out. This has been revised within the text to mirror the term used by discussants in the two quotes. The discussants referred to the disease name as ‘FMD’. This level of familiarity with the disease due to the vaccination campaigns seemed worth mentioning, so we also added an additional line of text about it. Lines 503-504

“The respondents were familiar enough with FMD to refer to it directly using the abbreviation as shown in the quotes below.”

**Comment:** Line 727: Maybe “investigated” or similar would be a more fitting verb than “asked”? Please consider rewording.

**Response:** This has been revised. Line 734

“This study investigated what men and women did to prevent or control diseases that affected them and their livestock as well as the factors that influenced the choice of disease control measures.”

**Comment:** Line 818-820: Please revise the sentence, now it is written to imply that women themselves are affected by PPR.

Response: This has been revised: Lines 849

“Given the low awareness of RVF and PPR, any recommendations about prevention and control were inferred from how people prevented or controlled other ruminant diseases.”

**Comment:** Line 858-861: This seems to be more of a summary than a conclusion. Please move to a more relevant section.

Response: This has been moved to the discussion section. Lines 836-840

Comment: Line 342-343: Please revise the sentence, the “minus the elderly” reads somewhat bizarre.

Response: Agreed, this has been deleted. Line 345

“We present the results of the thematic analysis in two sections: The first section uses GAF, and the second section uses SEM.”

Also added as a limitation from line 860.

“GAF structure includes the elderly for consideration as a separate category but there were not enough elderly respondents for separate analysis.”

Comment: Line 347-349: Please revise the sentence.

Response: This has been revised. Line 349

**Typological errors**

Line 5: A comma seems to be missing between the numbers for the affiliation 1 and 5.
Line 59: Please replace the comma after “fodder collection” with “and”.
Line 229: The sentence is not complete, please revise.
Line 252: Please replace the comma after “resource mapping” with “and”.
Line 259: Please remove “BUILD” from the sentence.
Line 339/Table 1: Please write out in the caption or as a foot note to the table what you mean by # (or write out “number of” in full).
Line 371: Please remove the comma after “isolation of sick animals”.
Line 381: Please remove the comma after “sick”.
Line 407 and other places: Please check how you mark the start of a new subsection (new line, indent, blank line) and use the same all through the manuscript.
Line 413-415: Please use italics for all citations, and use indent (or not) for all citations.
Line 458: Please remove the comma after “vendors”.
Line 497: Please write out numbers less than twelve in letters.
Line 524: Please remove the comma after “disease restrictions”.
Line 528: Please remove the comma after “lameness and death”.
Line 530: Please revise the sentence (remove the subclausation).
Line 619: Please place the reference at the end of the sentence.
Line 762: Please use the abbreviation (SR) once introduced.

Response: All the above comments highlighted on grammar and typos have been revised.

We look forward to your response and hope the revisions will enable you to accept this version for publication.

Sincerely,

Jane Namatovu, Peter Lule, Marsy Asindu, Zoë A. Campbell, Dan Tumusiime, Henry Kiara, Bernard Bett, Kristina Roesel, Emily Ouma
