# Supplementary material for: Gender roles in ruminant disease management in Uganda: Implications for the control of peste des petits ruminants and Rift Valley fever
Source: PLoS One. 2025 Apr 25;20(4):e0320991. doi: 10.1371/journal.pone.0320991 (PMC12027259; doi:10.1371/journal.pone.0320991)
Supplement: Attachment — Submitted filename: Response_to_Reviewers_auresp_6.docx [file pone.0320991.s011.docx]

Dr. Nussieba A. Osman

Academic Editor

PLOS ONE

February 27^th^, 2025

**Re: Revision required for manuscript PONE-D-24-01412R5**

Dear Dr. Osman,

We would like to thank the academic editor and the reviewer once again for taking the time to review our manuscript and for the helpful comments. We have addressed the comments raised and corrected some typographical errors.

These changes are detailed below.

**Editor’s Comments**:

Please review your reference list to ensure that it is complete and correct. If you have cited papers that have been retracted, please include the rationale for doing so in the manuscript text or remove these references and replace them with relevant current references.

Any changes to the reference list should be mentioned in the rebuttal letter that accompanies your revised manuscript. If you need to cite a retracted article, indicate the article’s retracted status in the References list and also include a citation and full reference for the retraction notice.

**Response**: Thanks very much for these valuable comments. We have rechecked the reference list thoroughly and updated the accession numbers for two references: Overholt et al (1985), reference 14 and Dione et al (2019), reference 74.

**Reviewer’s comments:**

**Comment:** Line 281: The information about Covid-19 is repetition and does not belong in this section.

**Response:** Thank you, the information about Covid-19 has been deleted from the text.

**Comment:** Line 325: I think that by “quotations” you might mean “quotes”?

**Response:** Thank you for the suggestion. This has been revised to quotes. Please refer to line 323.

**Comment:** Line 996: Please consider if the * after E should be removed in the reference list.

**Response:** Thank you for highlighting this. Reference 23 has been updated accordingly. Please refer to line 990.

**Comment**: Line 794: By “socio” do you mean “social”?

**Response:** Yes, this has been revised to social. Please refer to Line 788.

**Comment:** Line 244-246 and line 254-256: These two sentences seem to partly convey the same message, please consider merging.

**Response:** Thank you for the suggestion. This has been revised accordingly. Please refer to lines 252-254.

**Comment:** Line 369: I presume the number of ticks represent the number of FGDs in which at least one participant mentioned the respective measures? Can you please explain this in the foot note.

**Response**: Thank you. This has been revised to; the number of FGDs in which at least one participant mentioned the respective measure. Please refer to line 367.

**Comment:** Line 135, 390: I don’t think the author guidelines includes using an indent to start a new section. Please check and unify all through the manuscript.

**Response**: This has been unified through the manuscript.

**Comment**: Line 405-408: This is reported also in the introduction. Is it a result or background information? Please don’t duplicate.

**Response:** Thank you for pointing this out. We have retained this information only in the background. Please refer to lines 88-91.

**Typographical errors**

Line 55-56, 817: Please put the reference at the end of the sentence.
Line 162: Please replace the colon at the end of the sentence with a full stop.
Line 267-268: “by/via/using/over” seems to be missing before “telephone”.
Line 317: Please remove the space after the “/”.
Line 318: I think “participants” would read better than “their” here, please consider changing.
Line 826: Please remove “other”.

**Response:** We have revised all the above typographical errors

We look forward to your response and hope the revisions will enable you to accept this version for publication.

Sincerely,

Jane Namatovu, Peter Lule, Marsy Asindu, Zoë A. Campbell, Dan Tumusiime, Henry Kiara, Bernard Bett, Kristina Roesel, Emily Ouma
